# A Global Collaborative Discriminative Denoising Network for Text-to-Image Person Re-Identification

**DOI:** 10.3390/s26113604

**Published:** 2026-06-05

**Authors:** Shaozhen Han, Shuai Guo

**Affiliations:** 1 College of Science, Qingdao University of Technology, Qingdao 266000, China; 202305060168@stu.qut.edu.cn; 2 School of Information and Control Engineering, Qingdao University of Technology, Qingdao 266000, China

**Keywords:** Text-to-Image Person Re-Identification, cross-modal retrieval, fine-grained alignment, attention mechanism

## Abstract

Text-to-Image Person Re-Identification (TI-ReID) aims to retrieve target pedestrians from large-scale image galleries using natural language descriptions. Despite recent progress achieved by dual-tower architectures based on vision-language pre-training, these methods remain susceptible to semantic misalignment and noise induced by occlusions, background clutter, and fine-grained attribute distractions. To mitigate these issues, we propose a Global Collaborative Discriminative Denoising Network (GCDD), a dual-tower fine-tuning framework built upon a CLIP visual encoder and a BERT text encoder. Specifically, GCDD introduces three complementary branches for robust feature enhancement. First, Discriminative Token Selection (DTS) performs adaptive hard filtering to suppress low-informative tokens. Second, Global-Guided Feature Adaptation (GFA) leverages modality-specific global semantics to recalibrate local features. Third, Query-Driven Aggregation (QDA) constructs more discriminative global representations via attentive pooling, where the backbone global feature serves as the query. The outputs of the three branches are fused through a parameter-free averaging strategy to produce the final representation. Extensive experiments on three standard TI-ReID benchmarks demonstrate that GCDD achieves strong competitive performance, validating the effectiveness of the proposed feature enhancement framework.

## 1. Introduction

TI-ReID aims to retrieve corresponding pedestrian images from large-scale person image databases using natural-language descriptions. As a fine-grained cross-modal retrieval task, TI-ReID enables identity-oriented retrieval even when the query image of the target pedestrian is unavailable, thus complementing traditional image-based person re-identification and showing practical value in real-world scenarios such as public security and intelligent surveillance [1]. The core challenge of TI-ReID lies in the substantial modality gap between visual and textual representations. Moreover, discriminative identity cues are distributed across both local details, such as clothing attributes and accessories, and global semantic patterns, such as overall appearance and contextual semantics, making it difficult to establish stable, accurate, and semantically aligned cross-modal correspondences. These difficulties become more severe in complex scenes with background clutter, occlusion, and description ambiguity, further hindering robust cross-modal matching.

To reduce the discrepancy between image and text modalities, early work mainly developed along two lines: global matching and local alignment. Global matching methods [2,3,4,5,6] map visual and textual features into a shared embedding space, making them efficient at inference time. Yet, without explicitly modeling local attributes, they often struggle to distinguish between visually similar people. In comparison, local alignment methods [7,8,9,10,11,12] focus on learning finer correspondences between image regions and textual descriptions. This line of work covers both explicit local alignment methods, which commonly depend on external tools or predefined part segmentation, and implicit attention-based methods, which try to learn such correspondences automatically. However, explicit local methods are often limited by the quality of external models and can be easily affected by noise in occluded or low-resolution scenarios. Implicit attention-based methods, although more flexible, may still retain background distractions in complex scenes.

Recently, visual-language pre-training (VLP) models, particularly CLIP [13], have become the dominant backbone for TI-ReID owing to their strong semantic representation and cross-modal alignment capabilities. However, directly transferring CLIP to TI-ReID still faces evident task-specific gaps. Its pre-training objective is primarily designed for general semantic alignment, whereas TI-ReID requires identity-related fine-grained discriminative cues. To bridge this gap, prior studies have improved robustness through enhanced fine-grained modeling and more effective training strategies, including UFineBench [14], PLOT [15], DM-Adapter [16], BEAT [17], adaptive uncertainty learning [18], and local augmentation methods [19]. Despite steady progress achieved by approaches such as CFine [20] and IRRA [21], many existing methods still rely on soft attention or implicit feature weighting to suppress background interference. In complex scenarios, irrelevant visual regions may still participate in feature aggregation, weakening the discriminability and stability of cross-modal matching [22]. Meanwhile, recent studies have shown that pair-level noisy correspondences or incomplete image–text annotations can also undermine reliable cross-modal learning [23,24,25,26]. Different from these pair-level robust correspondence learning studies, this work focuses on token-level and feature-level noise suppression within each modality under the standard paired TI-ReID setting.

**Figure 1:**
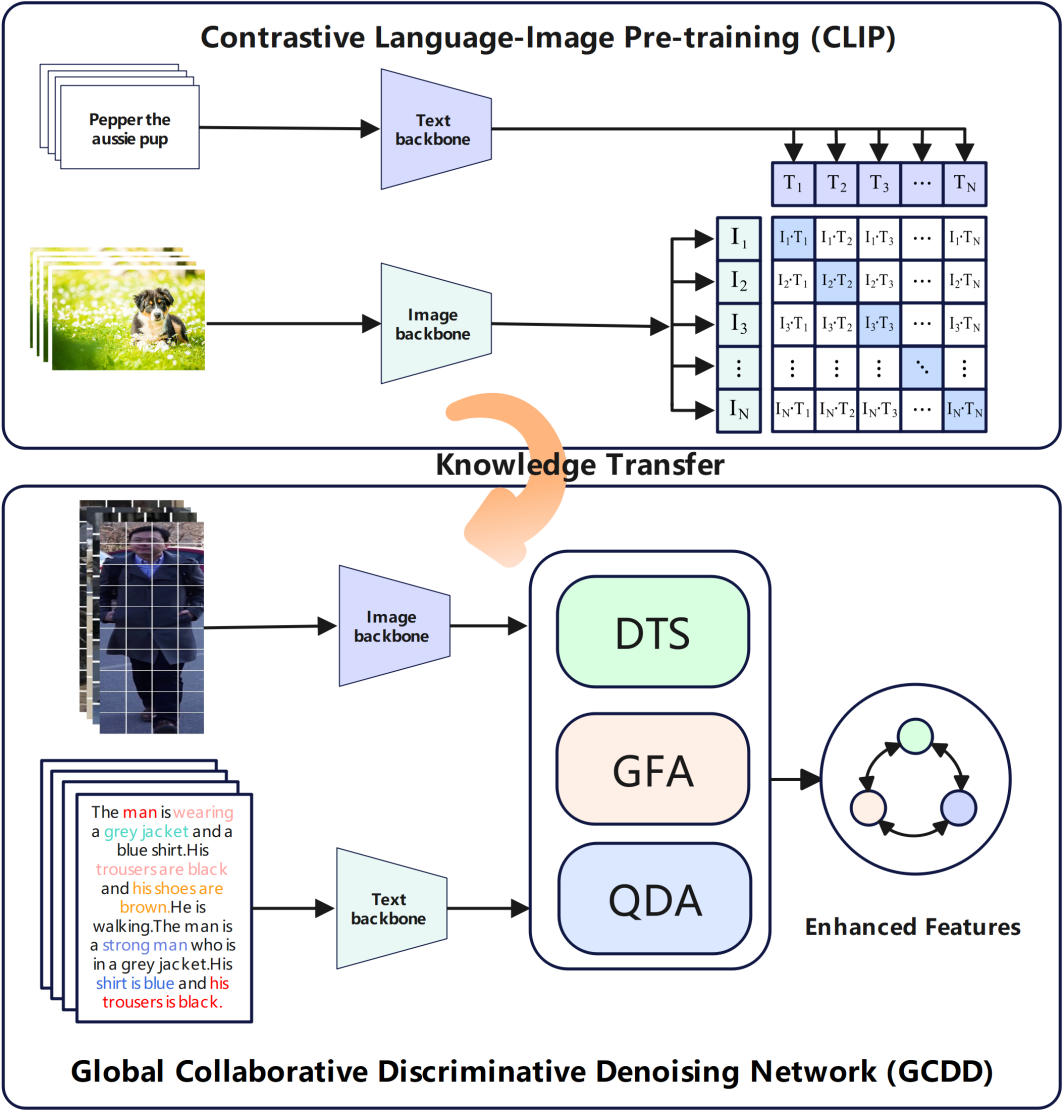
Comparison between the standard CLIP paradigm and the proposed GCDD framework. Standard CLIP-based approaches mainly rely on global representation matching, which may overlook fine-grained cues and remain sensitive to background clutter. Building on a CLIP-driven dual-encoder paradigm, GCDD introduces a symmetric discriminative framework consisting of DTS, GFA, and QDA to suppress irrelevant tokens and enhance fine-grained representation learning, yielding more robust and discriminative embeddings for TI-ReID.

Based on these observations, we propose a CLIP-driven Global Collaborative Discriminative Denoising Network (GCDD), whose overall architecture is illustrated in Figure 1. Under the dual-tower retrieval paradigm with independent encoding during inference, GCDD improves robustness through three complementary feature enhancement mechanisms. Specifically, DTS explicitly discards low-information background tokens to improve the feature signal-to-noise ratio. GFA recalibrates the local sequence using the global representation as a semantic anchor, alleviating local semantic misalignment and strengthening semantic consistency. QDA further performs query-driven attentive aggregation over contextualized local features to produce a more discriminative global representation for retrieval matching. Since these three branches enhance representations from different perspectives, we adopt a parameter-free averaging fusion strategy to combine their outputs. To avoid the high cost of token-level cross-modal interaction, GCDD confines these enhancements within each modality and performs retrieval during inference solely via representation-level cosine similarity, thereby remaining compatible with the standard dual-tower evaluation protocol and the independent encoding requirement of TI-ReID. The additional computation is mainly introduced by the intra-modal attention and aggregation enhancement layers, while the overall framework preserves the standard dual-tower retrieval pipeline and supports independent encoding of images and texts. The main contributions of this paper are as follows:

- We propose the GCDD framework, which improves the robustness of CLIP fine-tuning under background interference and fine-grained semantic misalignment through explicit denoising and a global guidance mechanism.
- We design the DTS, GFA, and QDA modules to enhance modality-specific representations without relying on external auxiliary tools, while preserving the inference compatibility of the dual-tower framework. The three modules adopt a parallel-branch architecture with parameter-free averaging fusion, and the modality-specific encoding paths maintain independent dual-tower inference.
- Experimental results on three public benchmark datasets (CUHK-PEDES [1], ICFG-PEDES [27], and RSTPReid [22]) show that the proposed method achieves competitive performance under multiple evaluation metrics.

The rest of this paper is structured as follows. Section 2 reviews the related work. Section 3 presents the proposed GCDD in detail. Section 4 reports the experimental results and corresponding analyses. Section 5 concludes the paper, and Section 6 discusses limitations and future work.

## 2. Related Work

### 2.1. Global Matching and Local Alignment for TI-ReID

TI-ReID, proposed by Li et al. [1], aims to retrieve corresponding pedestrian images from a gallery using natural language descriptions. Existing methods can be broadly categorized into three types: global matching methods, explicit local alignment methods, and implicit attention-based alignment methods. The first category comprises global matching methods. Early works such as [2,3] mainly align global image–text embeddings by learning joint latent spaces, such as cross-modal projection matching (CMPM) [2] or instance-level supervised matching loss [3]. These methods are computationally efficient, but because they rely solely on global representations, they often fail to capture fine-grained differences such as clothing textures and accessories, leading to limited discriminative ability. The second category comprises explicit local alignment methods. ViTAA [7] and PMA [8] explicitly align body parts using external priors such as feature parsing and pose estimation, while TIPCB [11] employs fixed strip segmentation to establish local correspondences. However, these approaches usually depend on the quality of external models or predefined segmentation rules, making them prone to noise and semantic disruption under occlusion, low resolution, or complex backgrounds. The third category comprises implicit attention-based alignment methods, including recent approaches such as IVT [28], SSAN [27], and a series of Transformer variants [29,30,31,32], which use attention mechanisms to automatically discover local correspondences without external tools. However, in practice, soft attention often only down-weights background regions rather than fully removing them. As a result, residual background clutter may still be retained in the learned representations and harm matching performance. Unlike these paradigms, our method explores more direct token-level filtering and representation learning from the perspective of discriminative detail modeling and noise suppression. By removing low-contribution background information and further enhancing fine-grained alignment through global guidance and multi-branch discriminative feature aggregation, the proposed method supports robust cross-modal retrieval while preserving the standard dual-tower retrieval pipeline.

### 2.2. Vision-Language Pre-Training for TI-ReID

Recently, the “pre-training-fine-tuning” paradigm has become dominant in cross-modal tasks. Early TI-ReID methods typically used unimodal backbones, such as ResNet [33] for images and BERT [34] for text. Since these backbones are pre-trained on different single-modal data sources, they lack inherent cross-modal correspondence and usually require additional downstream alignment training. Visual-language pre-training (VLP) models have alleviated this issue by learning transferable image–text representations from large-scale paired data. In particular, CLIP [13] learns strong image–text semantic alignment and has shown strong transferability. Given the limited scale and domain gaps of TI-ReID datasets, leveraging CLIP’s cross-modal prior knowledge is beneficial for improving retrieval robustness. With the success of CLIP, many studies have explored how to adapt VLP models to TI-ReID. Han et al. [35] first fine-tuned CLIP with momentum contrastive learning under limited-data settings. Later works mainly focus on two directions: fine-grained alignment and improved training or adaptation strategies. For fine-grained modeling, CFine [20] enhances matching through multi-level fine-grained feature extraction, while IRRA [21] improves visual–textual correspondence by implicit relation reasoning. UFineBench [14] provides ultra-fine-grained benchmarks for evaluating subtle attribute-level differences. PLOT [15] discovers corresponding pedestrian regions using part slot attention, and Zhang et al. [19] improve matching through local-enhanced representation learning. For training and adaptation strategies, BEAT [17] explores bidirectional one-to-many embedding alignment, DM-Adapter [16] introduces domain-aware mixture-of-adapters to handle data distribution variations, Li et al. [18] improve robustness through adaptive uncertainty-based learning, and CSKT [36] investigates CLIP-based synergistic knowledge transfer for text-based person retrieval by exploiting complementary knowledge from CLIP.

Although these CLIP-based and VLP-based methods have achieved notable progress, TI-ReID remains challenging in complex scenes with background clutter, occlusion, incomplete visual cues, and visually similar pedestrians. Many existing methods mainly rely on soft attention or implicit feature weighting to emphasize important regions or tokens. However, such soft mechanisms usually down-weight irrelevant information rather than explicitly removing it, so residual background or low-discriminative tokens may still participate in feature aggregation and affect cross-modal matching. In contrast, our GCDD framework focuses on intra-modal discriminative denoising and feature enhancement while preserving the dual-tower retrieval paradigm. Specifically, DTS performs adaptive hard token filtering to explicitly suppress low-information visual patches or textual tokens, GFA recalibrates full local sequences with modality-specific global guidance to improve semantic consistency while preserving useful details, and QDA constructs retrieval-oriented global representations through query-driven aggregation to emphasize discriminative local cues while maintaining independent image and text encoding during inference.

### 2.3. Noisy Correspondence and Partial Alignment Learning

In addition to background and token-level noise, another related research line focuses on noisy, incomplete, or partially reliable image–text correspondences. Qin et al. [23] explicitly studied noisy-correspondence learning for TI-ReID, where under-correlated or false-correlated image–text pairs may be incorrectly treated as positive pairs during training. PCCA [24] further considered incomplete text-based person re-identification, where image and text data may be partially missing or not completely matched, and introduced prototype-guided cross-modal completion and alignment. PASA [25] proposed partial-negative and soft-label alignment to alleviate unreliable hard-negative mining in text-to-image person retrieval. CANC [26] explored robust text–image person retrieval in open environments by addressing unlabeled and incomplete multimodal data through semantic alignment and neighbor-aware completion. These studies mainly aim to improve the reliability of image–text pair construction, incomplete-modality modeling, or supervision signals under imperfect correspondence conditions. In contrast, our GCDD assumes the standard supervised paired TI-ReID setting and focuses on intra-modal token-level feature enhancement after image–text pairs are given. Specifically, it suppresses background noise through discriminative token selection, refines local feature sequences under global semantic guidance, and further enhances retrieval representations via query-driven attentive aggregation. Therefore, GCDD is orthogonal and potentially complementary to pair-level noisy-correspondence learning, partial alignment learning, and incomplete-modality completion methods, while preserving the independent inference property of the dual-tower framework.

## 3. Methods

**Figure 2:**
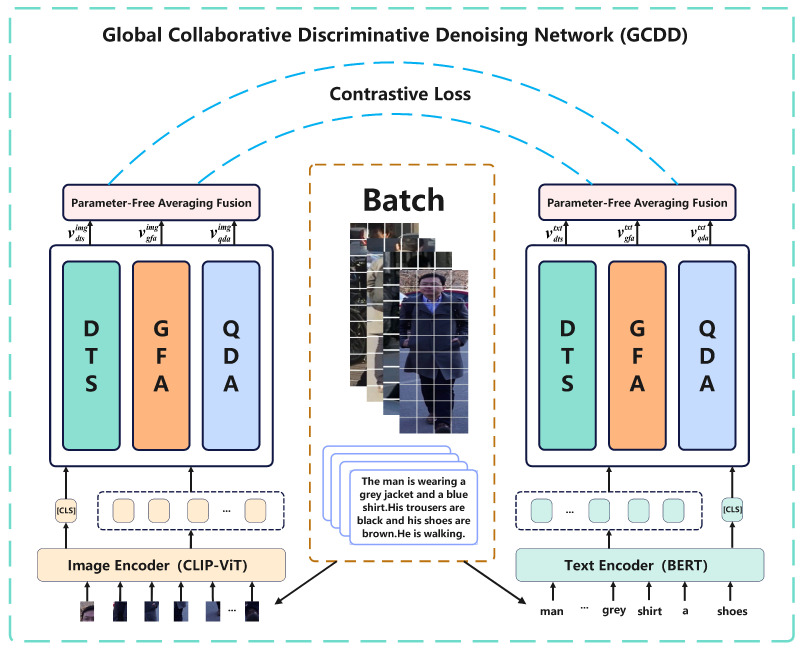
Overview of the GCDD model. The framework adopts a dual-tower architecture based on CLIP-ViT and BERT, symmetrically introducing three parallel enhancement branches for both modalities: (1) DTS suppresses low-information tokens through attention-based hard filtering. (2) GFA calibrates local features under global semantic guidance. (3) QDA adaptively aggregates discriminative information by using the global representation as the query. The outputs of the three branches are combined through parameter-free averaging without introducing additional learnable fusion parameters. The model is optimized solely with the bidirectional InfoNCE contrastive loss. During inference, each tower independently produces $L_{2}$-normalized representations for cosine-similarity-based retrieval.

In this section, we will provide a detailed description of the implementation specifics of our proposed GCDD. Figure 2 presents an overview of the GCDD implementation.

### 3.1. Dual-Encoder Feature Extraction

GCDD adopts a heterogeneous dual-encoder architecture consisting of a CLIP-pretrained visual encoder and a BERT-based textual encoder. The original CLIP model learns a shared image–text latent space through large-scale vision-language pre-training, which provides strong generic cross-modal alignment. However, directly transferring this representation space to TI-ReID remains challenging, since TI-ReID focuses on fine-grained identity-level matching rather than general semantic correspondence. In this task, textual descriptions usually contain detailed pedestrian attributes, such as clothing colors, accessories, body parts, carried objects, and subtle appearance cues, making token-level language modeling important for capturing discriminative textual information. To better adapt vision-language pre-training to fine-grained person retrieval, we retain the CLIP-pretrained ViT encoder [37] as the visual branch and employ BERT-base [34] as the textual branch. The visual encoder provides strong visual representation priors learned from large-scale vision-language data, while BERT offers contextual token-level representations for attribute-rich pedestrian descriptions. Compared with CLIP’s original text encoder, which is mainly optimized for global image-caption alignment, BERT is more suitable for modeling fine-grained attribute phrases through bidirectional contextual token representations. This property is consistent with the design of DTS, GFA, and QDA, which operate on token-level local representations for discriminative feature enhancement. This task-oriented encoder design is also related to prior TI-ReID studies such as CFine [20], which suggest that adapting CLIP to fine-grained person retrieval benefits from more task-specific representation modeling. In our framework, the heterogeneous visual and textual representations are further aligned during downstream TI-ReID fine-tuning. This design allows GCDD to balance the transferable visual prior of CLIP with the task-specific textual understanding required for fine-grained person retrieval. Moreover, after removing the projection layer, CLIP-ViT-B/16 produces 768-dimensional visual token features, and BERT-base naturally outputs 768-dimensional textual features. This provides a unified high-dimensional feature space for the subsequent DTS, GFA, and QDA modules without introducing an additional dimensionality-reduction projection.

Image Encoder. Given an input image $I\in R^{H\times W\times C}$, we employ a CLIP-pretrained Vision Transformer (ViT) as the image encoder. The image is first divided into $N_{v}=\left(H\times W\right)/P^{2}$ non-overlapping image patches, where *P* denotes the image block size. Each image patch is linearly projected onto a d-dimensional embedding space. A learnable [CLS] token is added to the beginning of the sequence to aggregate global image information. After positional embeddings are added, the token sequence is processed through a Transformer layer, $F^{img}=\mathrm{ViT}\;\left(\left[f_{cls}^{v};\;f_{1}^{v};\;f_{2}^{v};\;\ldots ;\;f_{N_{v}}^{v}\right]\right)\in R^{(N_{v}+1)\times d}$, where $f_{\mathrm{cls}}^{v}$ denotes the visual-side [CLS] token. The output of the image encoder is denoted by $F^{\mathrm{img}}=\left\{v_{g},v_{1},v_{2},\ldots ,v_{N_{v}}\right\}$, where $v_{g}\in R^{d}$ is the global representation derived from the [CLS] token, and $H^{\mathrm{img}}=\left\{v_{1},v_{2},\ldots ,v_{N_{v}}\right\}\in R^{N_{v}\times d}$ denotes the patch-level local representations.

Text Encoder. For the input text description S, we employ a pre-trained BERT [34] model as the text encoder to generate text representations. The text is first tokenized using the WordPiece tokenizer associated with the selected BERT model. We prepend [CLS] to the beginning of the sequence as a sentence-level representation and append [SEP] to the end as the sequence terminator. The resulting token sequence of length $N_{t}+2$ is then fed into the BERT Transformer, $F^{\mathrm{txt}}=\mathrm{BERT}\;\left(\left[f_{\mathrm{cls}}^{t};\;f_{1}^{t};\;f_{2}^{t};\;\ldots ;\;f_{N_{t}}^{t};\;f_{\mathrm{sep}}^{t}\right]\right)\in R^{(N_{t}+2)\times d}$, where $f_{\mathrm{cls}}^{t}$ denotes the textual-side [CLS] token, and $f_{\mathrm{sep}}^{t}$ denotes the [SEP] token. The text encoder produces $F^{\mathrm{txt}}=\left\{t_{g},t_{1},t_{2},\ldots ,t_{N_{t}}\right\}$, where $t_{g}\in R^{d}$ denotes the global representation derived from the [CLS] token, and $H^{\mathrm{txt}}=\left\{t_{1},t_{2},\ldots ,t_{N_{t}}\right\}\in R^{N_{t}\times d}$ denotes the corresponding token-level local representations. To unify the notation, we introduce the modality index m∈{img,txt}, where $g^{m}$ denotes the global [CLS] representation of modality *m*, with $g^{img}=v_{g}$ and $g^{txt}=t_{g}$.

Although the aforementioned dual-tower backbone network can extract fundamental visual and textual features, direct cross-modal retrieval using raw local sequences remains challenging. To address this, we introduce three parallel feature enhancement modules (DTS, GFA, and QDA) after the backbone network to mine fine-grained information. These modules work synergistically to reconstruct the raw backbone features into high-signal-to-noise-ratio global representations, enabling more precise cross-modal matching under the dual-tower retrieval setting.

### 3.2. Discriminative Token Selection

**Figure 3:**
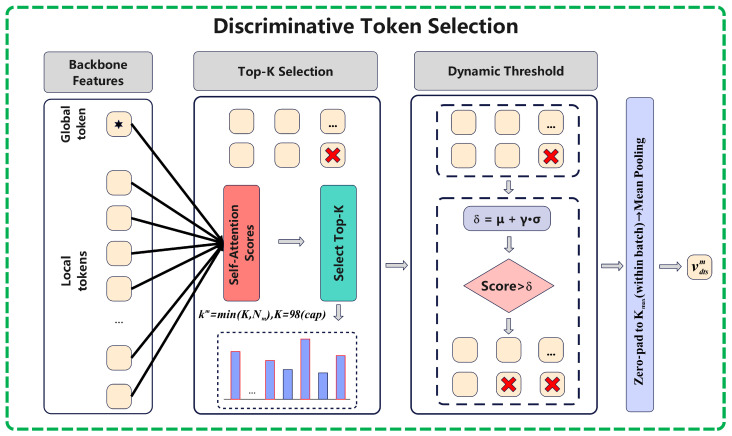
Discriminative token selection. Within the sequence self-attention mechanism, this module adopts a coarse-to-fine filtering strategy based on attention responses from global to local tokens. It first selects candidate tokens with high attention scores and then uses an adaptive threshold to suppress background and other irrelevant tokens, making identity-related cues more salient.

Cross-modal retrieval faces not only the challenge of modal heterogeneity but is also constrained by intra-modal asymmetric noise. Image background clutter and redundant textual modifiers significantly dilute core identity cues. Traditional soft attention mechanisms, being inherently dense-weighted, struggle to thoroughly eliminate residual low-weight noise, thereby compromising subsequent feature aggregation and alignment. Inspired by hard attention [38], we propose a DTS mechanism that shifts noise suppression from soft weight recalibration to the structural screening stage. By explicitly hard-filtering out background or weakly correlated tokens within the DTS branch, we suppress their influence. Within the three-branch parallel framework, the remaining branches retain the full sequence as a safety net for information, balancing noise reduction with data integrity. This module is symmetrically embedded into the dual-tower branches, with the overall workflow illustrated in Figure 3. We directly utilize the token sequence output from the backbone network as input: $F^{m}=\left[f_{0}^{m};\;f_{1}^{m};\;\ldots ;\;f_{N_{m}}^{m}\right]\in R^{(N_{m}+1)\times d}$, where $f_{0}^{m}$ denotes the global token, corresponding to the [CLS] token of the ViT on the image side and the [CLS] token of BERT on the text side. ${\left\{f_{t}^{m}\right\}}_{t=1}^{N_{m}}$ on the text side specifically refers to token-level features after excluding the [SEP] token, and *d* denotes the hidden dimension. For ViT-B/16, $N_{v}=196$ on the image side. $N_{t}$ on the text side depends on the input text length.

Learnable saliency scores. The first step in DTS is to accurately assess each local token’s contribution to the global identity semantics. Instead of directly using the raw attention maps from the backbone network, we introduce a lightweight, learnable self-attention layer on top of the backbone outputs, with parameters independent of the backbone. This layer re-encodes the input sequence using a standard multi-head self-attention mechanism. It not only produces attention responses for saliency estimation, but also outputs a set of re-encoded token representation sequences for subsequent selection and aggregation, thereby ensuring that this scoring module can be learned end-to-end under the downstream TI-ReID objective. Through this learnable self-attention layer, we obtain the re-encoded sequence ${\tilde{F}}^{m}=\left[{\tilde{f}}_{0}^{m};{\tilde{f}}_{1}^{m};\ldots ;{\tilde{f}}_{N_{m}}^{m}\right]$, and compute the multi-head attention responses from the global anchor ${\tilde{f}}_{0}^{m}$ to the local sequence. The saliency score $s_{t}^{m}$ is defined as the average response weight of the global token to the local token in the multi-head attention map:

(1) $$s_{t}^{m}=\frac{1}{n_{h}}\sum_{h=1}^{n_{h}}A_{0,t}^{\left(m,h\right)},\;t=1,\ldots ,N_{m},$$

where $A_{\left(0,t\right)}^{\left(m,h\right)}$ denotes the attention weight from the global token to the local token *t* in the *h*-th head, and $n_{h}$ is the number of attention heads. Since the global token ${\tilde{f}}_{0}^{m}$ has already aggregated the overall identity semantics of the sample, the response intensity computed with it as the semantic anchor can reflect the importance of the local position *t*, thereby providing an interpretable and stable discriminative basis for subsequent hard selection.

Additionally, for text modalities, we introduce a masking mechanism that sets the saliency scores of padding positions to negative infinity, thereby excluding them from the ranking phase. It is worth noting that the subsequent Top-*k* filtering and threshold-based decisions are both discrete selection operations, for which gradients are not computed with respect to the index selection process itself during backpropagation. However, since the selected token representations are derived from the differentiable re-encoded sequence ${\tilde{F}}^{m}$, the gradients of the downstream TI-ReID loss with respect to the selected token features can still be effectively backpropagated to the learnable parameters of this self-attention layer. This design enables the saliency scoring criteria to be driven by downstream identity recognition objectives throughout the end-to-end training process, thereby achieving adaptive optimization of scoring standards and continuous enhancement of task relevance.

Two-stage adaptive hard filtering. After obtaining the saliency scores, the core challenge is to set an appropriate threshold to separate foreground and background regions effectively. Due to significant variations in foreground coverage across different samples, fixed-threshold strategies often result in critical information loss or residual noise. To address this, we design a two-stage adaptive hard filtering strategy:

(1) Preliminary screening stage. Retain the Top-$k_{m}$ high-response tokens to form the candidate set $C^{m}$. Considering the intrinsic difference in length scales between image and text sequences, and that reusing the fixed candidate upper bound from the image branch on the text side may degenerate into full retention under short-sequence settings, we separately specify the coarse-screening retention scale for the two modalities. Specifically, the image branch adopts a fixed-ratio upper bound $K_{\mathrm{cap}}^{\mathrm{img}}=\left\lfloor K_{\mathrm{ratio}}^{\mathrm{img}}\cdot N_{v}\right\rfloor$, while the text branch adopts an adaptive ratio upper bound $K_{\mathrm{cap}}^{\mathrm{txt}}=\max\;\left(1,\;\left\lfloor K_{\mathrm{ratio}}^{\mathrm{txt}}\cdot N_{t}\right\rfloor \right)$. Accordingly, the number of items retained after coarse screening is defined as $k_{m}=\min\;\left(K_{\mathrm{cap}}^{m},\;N_{m}\right)$, where $K_{\mathrm{cap}}^{m}$ takes $K_{\mathrm{cap}}^{\mathrm{img}}$ or $K_{\mathrm{cap}}^{\mathrm{txt}}$ for different modalities. The corresponding candidate set is:

(2) $$C^{m}=\mathrm{Top}\text{-}k_{m}\;\left({\left\{s_{t}^{m}\right\}}_{t=1}^{N_{m}}\right).$$

To prevent selection degradation in extreme cases, we implement a forced retention mechanism that ensures each sample retains at least one token with the highest response. Furthermore, within the three-branch parallel framework during training, the other branches that maintain full-sequence input serve as backup sources of information, thereby ensuring the completeness and robustness of feature learning.

(2) Fine-grained screening stage. Within the candidate set, we exploit statistical characteristics to adaptively determine the truncation boundary. Let the set of candidate scores be $\left\{\;s_{t}^{m}|t\in C^{m}\;\right\}$, whose mean and standard deviation are denoted by $\mu^{m}$ and $\sigma^{m}$, respectively. The dynamic threshold is defined as:

(3) $$\delta^{m}=\mu^{m}+\gamma \sigma^{m},$$

where γ controls the screening sensitivity. Since the threshold is adaptively determined by the mean and variance of candidate set scores, this strategy automatically adjusts the cutoff boundary across different samples, prioritizing the retention of relatively more prominent high-response tokens. The final retained set is:

(4) $$P^{m}=\left\{\;t\in C^{m}\;|\;s_{t}^{m}>\delta^{m}\;\right\},$$

where $P^{m}$ denotes the index set of tokens retained in the final selection. If an extreme case occurs where no token in a sample passes the threshold, fall back to retaining the token with the highest score in the candidate set to ensure subsequent aggregation can always proceed.

Batch-wise Dynamic Padding and Masked Mean Pooling. After the two-stage filtering, the *i*-th sample under modality *m* yields the final retained token index set $P_{i}^{m}$, whose length is $L_{i}=\left|P_{i}^{m}\right|$. To facilitate batch processing and maintain consistent tensor shapes, we take the maximum retained length within the same batch as $K_{\max}=\max_{i}\left|P_{i}^{m}\right|$. Subsequently, we collect the retained token features for each sample from the re-encoded sequence ${\tilde{F}}^{m}$ and pack them in order into the first $L_{i}$ positions; the remaining positions are padded with zero vectors up to $K_{\max}$, thereby obtaining the aligned representation $H_{\mathrm{dts}}^{m}\left(i\right)\in R^{K_{\max}\times d}$. Meanwhile, we maintain a lengthwise binary mask $M_{i}\in {\left\{0,1\right\}}^{K_{\max}}$, where $M_{i}\left(j\right)=1$ if $j\leq |P_{i}^{m}|$, and $M_{i}\left(j\right)=0$ otherwise. A fallback mechanism enforces $|P_{i}^{m}|\geq 1$, thereby avoiding degenerate cases in which an empty sequence would render subsequent aggregation infeasible. To prevent zero-padding from interfering with sequence aggregation, we employ a masked weighted averaging strategy to obtain the DTS representation vector for each sample:

(5) $$v_{\mathrm{dts}}^{m}\left(i\right)=\frac{\sum_{j=1}^{K_{\max}}M_{i}\left(j\right)\cdot H_{\mathrm{dts}}^{m}\left(i,j\right)}{\sum_{j=1}^{K_{\max}}M_{i}\left(j\right)+\epsilon },$$

where ϵ is a numerical stabilizer, used to prevent a zero denominator and to ensure that the aggregated representation is determined solely by valid tokens. This strategy facilitates batch processing while stably yielding accurate feature representations.

### 3.3. Global-Guided Feature Adaptation

**Figure 4:**
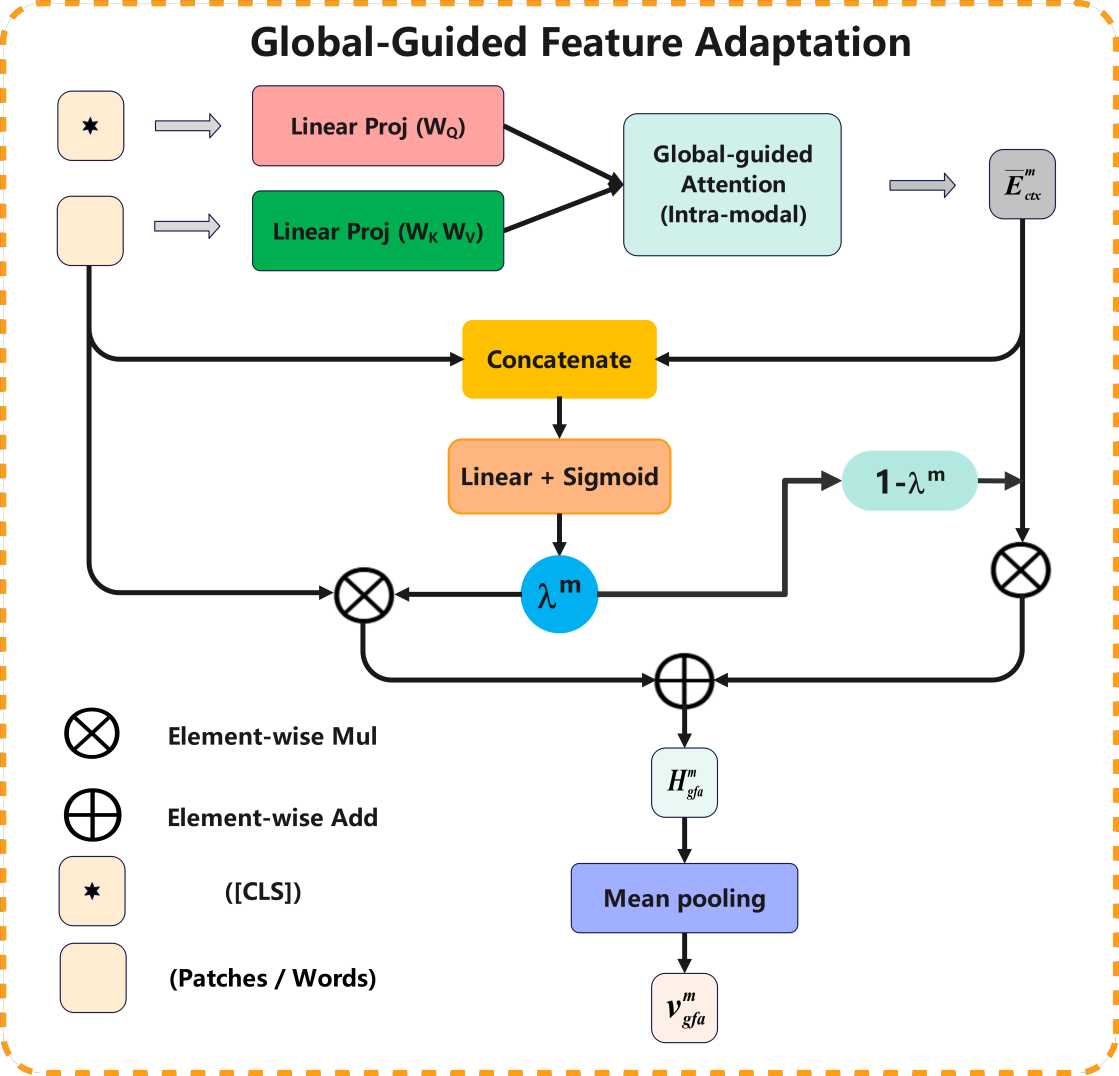
Global-guided feature adaptation. The backbone global representation is used as a semantic anchor to perform globally guided intra-modal attention over the full local sequence, producing a globally attended context representation. This shared context is then injected into local tokens through a position-wise gated fusion mechanism, which adaptively balances original local details and global contextual information to obtain a recalibrated full-length sequence representation. Finally, mean pooling is applied to derive the branch representation for retrieval.

Although the two-stage hard filtering strategy in DTS is effective for reducing background noise, its discrete token selection may interrupt local contextual continuity. In visual sequences, fine-grained identity cues are often spread across spatially separated regions, and removing intermediate tokens may weaken the semantic relationship among them. In textual sequences, attribute expressions often depend on syntactic structure and long-range token dependencies, which may likewise be affected by hard selection. To mitigate this problem, we introduce the GFA module as a parallel branch to complement DTS. Instead of directly discarding tokens, GFA extracts a globally attended context signal from the complete local sequence and injects it back into each position through adaptive gated fusion. This allows GFA to preserve sequence integrity while improving global semantic consistency, thereby serving as a complement to DTS. The overall architecture of GFA is shown in Figure 4.

Dual-Tower Architecture Constraints. Unlike cross-modal attention mechanisms, GFA operates strictly within each modality: the image global representation guides only the image local sequence, and the text global representation guides only the text local sequence. This design avoids explicit cross-modal feature mixing and preserves the independent encoding property required by practical dual-tower retrieval.

Global-Local Interaction Attention. Let the backbone local sequence be $H^{m}\in R^{N_{m}\times d}$, where m∈{img,txt}, and let the corresponding global representation be $g^{m}\in R^{d}$. The input of GFA is taken directly from the backbone local sequence rather than from the output of DTS. We first project the local sequence and the global representation into the same hidden space:

(6) $${\tilde{H}}^{m}=\phi_{l}\left(H^{m}\right)\in R^{N_{m}\times d},\;q^{m}=\phi_{q}\left(g^{m}\right)\in R^{d},$$

where $\phi_{l}$ and $\phi_{q}$ denote the local-sequence projection and global-query projection, respectively.

We then use the projected global representation as a modality-specific semantic anchor to attend over the projected local sequence and obtain a globally attended context vector:

(7) $$e_{\mathrm{ctx}}^{m}=\mathrm{MHA}\;\left(q^{m},{\tilde{H}}^{m},{\tilde{H}}^{m}\right)\in R^{d}.$$

For the text branch, a padding mask is applied to exclude invalid tokens. In implementation, the global query may be expanded along the sequence dimension before calling multi-head attention; since the expanded queries are identical across positions, this is mathematically equivalent to computing a single context vector and then broadcasting it for subsequent fusion. To match the sequence length, the context vector is broadcast along the token dimension:

(8) $${\bar{E}}_{\mathrm{ctx}}^{m}=\mathrm{Expand}\;\left(e_{\mathrm{ctx}}^{m}\right)\in R^{N_{m}\times d}.$$

In this way, the global representation summarizes globally relevant information from the complete local sequence while preserving the original token structure.

Adaptive Gate Fusion. Directly replacing each position of the original local sequence with the same globally attended context may oversmooth fine-grained local details. To balance global semantic consistency and local detail preservation, we introduce a position-wise adaptive gate:

(9) $$\lambda^{m}=\sigma \;\left(\left[H^{m},{\bar{E}}_{\mathrm{ctx}}^{m}\right]W_{\mathrm{gate}}\right)\in R^{N_{m}\times d},$$

where σ(·) denotes the sigmoid function, $W_{\mathrm{gate}}\in R^{2d\times d}$ is the gating projection matrix, and [·,·] denotes feature concatenation. The recalibrated sequence is then obtained by:

(10) $$H_{\mathrm{gfa}}^{m}=\lambda^{m}⊙H^{m}+\left(1-\lambda^{m}\right)⊙{\bar{E}}_{\mathrm{ctx}}^{m},$$

where ⊙ denotes element-wise multiplication. This mechanism allows each position to adaptively balance original local detail and globally guided contextual information. Notably, the position-specific variation in $H_{\mathrm{gfa}}^{m}$ is introduced by the adaptive gate rather than by the attended context itself.

The primary output of GFA is the recalibrated full-length sequence $H_{\mathrm{gfa}}^{m}$ rather than a compact retrieval vector, which distinguishes it from retrieval-oriented aggregation modules such as QDA. For branch-level representation learning, we further apply mean pooling over $H_{\mathrm{gfa}}^{m}$ to obtain the GFA branch representation $v_{\mathrm{gfa}}^{m}$. For the text modality, mean pooling is performed only over valid tokens to avoid dilution caused by padding.

### 3.4. Query-Driven Aggregation

**Figure 5:**
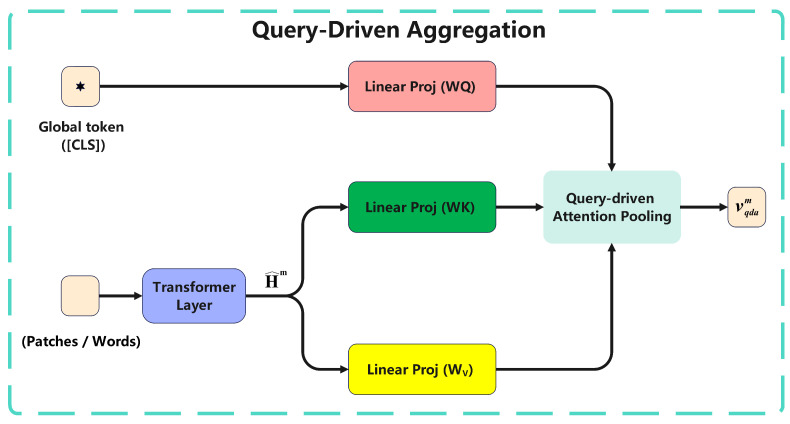
Query-driven aggregation. This module first employs a lightweight Transformer to model contextual dependencies within the local sequence. It then uses the corresponding global representation as the query to perform attention-based aggregation over the augmented local tokens. This process adaptively aggregates the most informative local cues to generate a compact global representation.

Although DTS and GFA significantly improve feature quality, both ultimately rely on mean pooling to generate global representations, implicitly assuming equal contributions from all spatial locations. However, this assumption is often overly simplistic in TI-ReID, where different locations still contribute unequally to identity recognition even after filtering or recalibration. For example, facial regions are typically more discriminative than leg regions, whereas simple averaging cannot capture such unevenly distributed discriminative cues. Inspired by the Object Query mechanism in DETR for object detection [39], we propose the QDA module. Unlike GFA, which preserves and refines the full local sequence, QDA is not intended to output another recalibrated sequence. Instead, it directly produces a single compact retrieval-oriented representation. Specifically, it abandons passive averaging in favor of an active identity-query mechanism, using the backbone global representation as a query to aggregate local features while dynamically extracting the most informative cues from local sequences for fine-grained TI-ReID. This process can be viewed as actively exploring which positions in the sequence best represent identity and automatically learning the optimal feature combination through attention. The overall architecture of the QDA module is illustrated in Figure 5.

Contextual Encoder. Before performing query-driven aggregation, we feed the local sequence features $H^{m}$ output by the backbone into a lightweight Transformer encoder to model long-range dependencies among tokens:

(11) $${\hat{H}}^{m}=\mathrm{TransformerEncoder}\;\left(H^{m}\right),$$

where, for the text branch, a padding mask is used to exclude invalid tokens, and the TransformerEncoder employs multi-head self-attention to encode the local sequence contextually. The encoder ensures that each token incorporates its surrounding context before aggregation, avoiding the isolated assessment of an individual token’s importance.

Query-driven attention pooling. We take the global representation $g^{m}$ of the backbone as the query vector $q^{m}$, which encodes the overall identity semantics of the sample. QDA performs aggregation via global-query-based attentive pooling, where the query is $q^{m}$ and the key and value are the contextually encoded sequence ${\hat{H}}^{m}$. Unlike GFA, which preserves the full token sequence as its primary output, QDA directly compresses the contextualized local sequence into a single retrieval-oriented vector through attention pooling. To maintain scale consistency in the feature space, we apply the scaling factor $1/\sqrt{d}$ when computing attention scores. We first perform linear projections:

(12) $$Q=q^{m}W_{Q},\;K={\hat{H}}^{m}W_{K},\;V={\hat{H}}^{m}W_{V},$$

where $W_{Q}$, $W_{K}$, and $W_{V}$ are learnable projection matrices that are independently instantiated for each modality m∈{img,txt} and do not share parameters. We then compute the attention fusion weights and perform aggregation:

(13) $${\mathrm{AttnWeights}}^{m}=\mathrm{Softmax}\;\left(\frac{QK^{⊤}}{\sqrt{d}}\right)\in R^{1\times N_{m}},$$

(14) $$v_{\mathrm{qda}}^{m}={\mathrm{AttnWeights}}^{m}\cdot V\in R^{d},$$

where $v_{\mathrm{qda}}^{m}$ denotes the aggregated global representation. For the text modality, padding positions are masked out before computing the attention weights, ensuring that only valid tokens contribute to the aggregation. The resulting global vector $v_{\mathrm{qda}}^{m}$ is a weighted combination of local features, where the weights are entirely determined by the response strength of local features to the query vector. Therefore, the output of QDA is not a refined sequence but the single aggregated vector $v_{\mathrm{qda}}^{m}$ used for retrieval-oriented matching. This procedure can be viewed as a form of single-query attention pooling, enabling the model to suppress irrelevant local cues and emphasize discriminative ones, thereby producing a more discriminative global representation.

Complementarity with DTS/GFA. QDA forms an organic, complementary relationship with the preceding two modules. Specifically, DTS performs hard filtering to suppress low-information or noisy tokens at the token level. GFA refines the full sequence through gated fusion and outputs a recalibrated sequence representation, while QDA aggregates key discriminative information through attention pooling and directly produces a compact vector for retrieval. Together with DTS, these three components handle the balance between noise suppression and feature preservation from different angles. In particular, GFA focuses on refining and preserving the full sequence before pooling, whereas QDA focuses on summarizing discriminative information for final matching. Since they differ in both output form and functional role, the two branches are complementary rather than redundant. Moreover, all three modules work independently on the same base features without cascading dependencies. They produce representations at different granularities, including a sparse discriminative subset, a semantically refined full sequence, and a compact global vector, which together offer complementary cues for the final parameter-free averaging fusion.

### 3.5. Three-Branch Parameter-Free Averaging Fusion

The DTS, GFA, and QDA branches enhance feature representations from different yet related perspectives. Specifically, DTS focuses on discriminative token filtering, GFA refines the full local sequence under global semantic guidance, and QDA produces a compact retrieval-oriented representation through query-driven attentive aggregation. Since these three branches enhance modality-specific representations in different ways, we adopt a simple parameter-free averaging strategy to combine their outputs.

For the *i*-th sample of modality *m*, the three branches produce branch-specific global representations, denoted as $v_{\mathrm{dts}}^{m}\left(i\right)\in R^{d}$, $v_{\mathrm{gfa}}^{m}\left(i\right)\in R^{d}$, and $v_{\mathrm{qda}}^{m}\left(i\right)\in R^{d}$, respectively. The final global representation is computed as:

(15) $$z_{\mathrm{final}}^{m}\left(i\right)=\frac{1}{3}\left(v_{\mathrm{dts}}^{m}\left(i\right)+v_{\mathrm{gfa}}^{m}\left(i\right)+v_{\mathrm{qda}}^{m}\left(i\right)\right).$$

This fusion strategy avoids introducing additional learnable fusion parameters while providing a straightforward way to integrate the complementary cues captured by the three branches. No additional branch-wise reweighting or normalization is applied before fusion. Since the fusion is performed entirely within each modality without requiring token-level cross-modal interaction, it also preserves the inference compatibility of the dual-tower framework.

### 3.6. Training and Inference

After parameter-free averaging of the three branch outputs, the final image and text representations in a mini-batch are denoted by ${\left\{z_{\mathrm{final}}^{\mathrm{img}}\left(i\right)\right\}}_{i=1}^{B}$ and ${\left\{z_{\mathrm{final}}^{\mathrm{txt}}\left(j\right)\right\}}_{j=1}^{B}$, respectively. Supervision is imposed only on these fused representations, without introducing auxiliary losses for individual branches. In this way, the DTS, GFA, and QDA branches are jointly optimized under a single retrieval objective.

Training Objective. We train the model with a bidirectional InfoNCE loss [40]. Given a mini-batch of *B* aligned image–text pairs, the *i*-th image and the *i*-th text form a positive pair, while all other cross-modal combinations within the mini-batch are treated as negatives. Let ${\hat{z}}_{\mathrm{final}}^{\mathrm{img}}\left(i\right)$ and ${\hat{z}}_{\mathrm{final}}^{\mathrm{txt}}\left(j\right)$ denote the $L_{2}$-normalized final image and text representations, respectively. Their pairwise cosine similarity is defined as:

(16) $$s_{ij}={\left({\hat{z}}_{\mathrm{final}}^{\mathrm{img}}\left(i\right)\right)}^{⊤}{\hat{z}}_{\mathrm{final}}^{\mathrm{txt}}\left(j\right),\;i,j=1,\ldots ,B,$$

where *B* denotes the mini-batch size. The image-to-text and text-to-image contrastive losses are defined as:

(17) $$L_{i2t}=-\frac{1}{B}\sum_{i=1}^{B}\log\frac{\exp\;\left(s_{ii}/\tau \right)}{\sum_{j=1}^{B}\exp\;\left(s_{ij}/\tau \right)},$$

(18) $$L_{t2i}=-\frac{1}{B}\sum_{j=1}^{B}\log\frac{\exp\;\left(s_{jj}/\tau \right)}{\sum_{i=1}^{B}\exp\;\left(s_{ij}/\tau \right)}.$$

The overall training objective is given by:

(19) $$L_{\mathrm{main}}=\frac{1}{2}\left(L_{i2t}+L_{t2i}\right).$$

In practice, label smoothing with a smoothing factor of ϵ=0.1 is applied when optimizing the bidirectional InfoNCE loss. This slightly softens the one-hot matching targets in the cross-entropy computation and encourages smoother similarity distributions between image and text representations.

Inference. During inference, the image and text towers operate independently, and no token-level cross-modal interaction is required. For each input sample, the DTS, GFA, and QDA branches produce three branch-specific global representations, which are combined by simple parameter-free averaging to obtain the final embedding. After $L_{2}$ normalization, cosine similarity is used for retrieval. Since gallery images can be encoded and stored offline, while text queries are processed independently online, the proposed method preserves the standard dual-tower retrieval pipeline.

## 4. Experiments

To validate the effectiveness of our method, we conduct experiments on three widely used TI-ReID benchmarks: CUHK-PEDES [1], ICFG-PEDES [27], and RSTPReid [22]. CUHK-PEDES is one of the earliest and largest benchmarks in this field, containing 40,206 images and 80,412 text descriptions for 13,003 pedestrians. Each image is manually annotated with two sentences of at least 23 words on average. Following the official split, the training, validation, and test sets contain 34,054, 3078, and 3074 images, as well as 68,108, 6156, and 6148 text descriptions, corresponding to 11,003, 1000, and 1000 identities, respectively. ICFG-PEDES contains 54,522 images of 4102 pedestrians, each paired with one text description. The average description length is 37 words, and the vocabulary contains 5554 unique words. Compared with CUHK-PEDES, its descriptions place greater emphasis on identity-related attributes and provide finer-grained details. According to the standard split, the training and test sets contain 34,674 and 19,848 image–text pairs from 3102 and 1000 identities, respectively. RSTPReid is designed for more challenging real-world scenarios. It contains 20,505 pedestrian images and 41,010 textual descriptions from 4101 identities collected across 15 cameras. Each identity is associated with five images, and each image is paired with two natural-language descriptions averaging about 23 words. Following the official split, the dataset is divided into 3701 identities for training, 200 for validation, and 200 for testing. These three benchmarks differ in scale, annotation granularity, and split settings, providing a comprehensive evaluation platform for TI-ReID methods under diverse scenarios. Detailed statistics are summarized in Table 1. For evaluation, we adopt Recall at Rank *K* (Rank-*K*, higher is better) and report Rank-1, Rank-5, and Rank-10 accuracy.

Implementation Details. All experiments are implemented in PyTorch 2.7.1 on a single NVIDIA RTX 4060 GPU. Input images are resized to 224×224, the maximum text length is set to 77, and the feature dimension is unified to d=768 for both modalities. CLIP-ViT-B/16 and BERT-base are adopted as the visual and textual encoders, respectively. In DTS, token-level discriminative scores are estimated by a 12-head self-attention module. The coarse screening ratios are set to $K_{\mathrm{ratio}}^{\mathrm{img}}=K_{\mathrm{ratio}}^{\mathrm{txt}}=0.5$, such that the top 50% tokens ranked by CLS-attention scores are selected as candidates, followed by dynamic thresholding with γ=1.0, yielding a sample-dependent number of retained tokens. GFA employs modality-specific 12-head multi-head attention with sigmoid gating and a dropout rate of 0.1. QDA consists of a single-layer Transformer encoder with 12-head attention, a 4096-dimensional feed-forward network, and GELU activation, followed by single-query attention pooling parameterized by modality-specific projection matrices $W_{Q}$, $W_{K}$, and $W_{V}$, with a scaling factor of $1/\sqrt{d}$. The three branches are fused by parameter-free averaging. Training is performed using the bidirectional InfoNCE objective with temperature τ=0.07. Label smoothing is applied to the bidirectional InfoNCE loss with a smoothing factor of ϵ=0.1. We use AdamW [41] with a layer-wise learning-rate schedule: the pretrained backbone encoders use a base learning rate of $2.2\times 10^{-5}$, while DTS, GFA, and QDA use a 7.5× higher learning rate of $1.65\times 10^{-4}$. Bias parameters use a 2× learning-rate multiplier without weight decay, while all other parameters use a weight decay of $10^{-4}$. The learning rate is linearly warmed up from 1% of the target value over the first 5 epochs and then decayed to $10^{-6}$ via cosine annealing. The model is trained for 50 epochs with a per-step batch size of 64, and gradients are accumulated over two steps before each optimizer update. Gradient clipping with a maximum norm of 1.0 is used during optimization. All main experiments are conducted with a fixed random seed of 42 for reproducibility. In addition, to examine the stability of the final GCDD model, we further conduct additional training and evaluation runs on CUHK-PEDES using two other random seeds, i.e., 123 and 2024. Together with the original seed-42 run, these results are used to compute the mean and standard deviation across three seeds, while all other settings are kept unchanged.

### 4.1. Comparison with Existing Methods

We compare GCDD with representative methods on three standard TI-ReID benchmarks, as shown in Table 2, Table 3 and Table 4. Overall, GCDD achieves strong and competitive performance across the three datasets. To evaluate its cross-backbone generalization ability, we further introduce two encoder variants, Ours-IN-ViT and Ours-CLIP-T, by replacing the CLIP visual encoder with an ImageNet-pretrained ViT and replacing the BERT text encoder with the original CLIP text encoder, respectively. These variants examine whether DTS, GFA, and QDA remain effective under different visual or textual backbones. The results provide preliminary evidence that GCDD is not restricted to a single CLIP-BERT configuration, but can generalize to alternative backbone architectures while maintaining competitive retrieval performance.

(1) Evaluation on CUHK-PEDES. We first evaluate GCDD on the widely used CUHK-PEDES dataset, and the results are reported in Table 2. GCDD achieves 68.30%, 85.56%, and 91.03% on Rank-1, Rank-5, and Rank-10, respectively. Although CSKT [36] obtains a slightly higher Rank-1 accuracy, GCDD achieves better Rank-5 and Rank-10 results, improving them from 84.93% to 85.56% and from 90.25% to 91.03%, respectively. This indicates that GCDD provides more stable retrieval performance at higher ranks, which is beneficial in practical retrieval scenarios where users often inspect multiple top-ranked candidates. Compared with early global matching methods such as Dual Path [3] and CMPM [2], as well as local modeling or attention-based methods, including DSSL [22], SSAN [27], and TIPCB [11], GCDD achieves clear improvements. In particular, it outperforms TIPCB by 4.98%, 2.64%, and 1.99% on Rank-1, Rank-5, and Rank-10, respectively, demonstrating the effectiveness of the proposed feature enhancement strategy. The two encoder variants further provide evidence for the backbone adaptability and generalization ability of the proposed framework. When the CLIP visual encoder is replaced with an ImageNet-pretrained ViT, Ours-IN-ViT still achieves 62.10%, 82.41%, and 88.01% on Rank-1, Rank-5, and Rank-10, respectively, remaining competitive with several non-CLIP-based methods. This indicates that DTS, GFA, and QDA are not strictly tied to the CLIP visual backbone and can still enhance representations under an alternative ImageNet-pretrained visual encoder. Similarly, Ours-CLIP-T obtains 66.38%, 80.54%, and 86.81%, showing that the framework can also be instantiated with a different textual encoder. Nevertheless, the full CLIP-BERT-based GCDD achieves the best overall performance among our variants, suggesting that this configuration is more suitable for fine-grained TI-ReID. Overall, these results show that GCDD not only benefits from the adopted CLIP-BERT configuration but also exhibits promising adaptability to different backbone choices. To examine the stability of GCDD under different random seeds, the main result is obtained with seed 42, and two additional runs are conducted on CUHK-PEDES using seeds 123 and 2024. Together with the original seed-42 run, these results form a three-seed stability analysis. The mean and standard deviation of Rank-1/Rank-5/Rank-10 are 68.35% ± 0.22%, 85.16% ± 0.36%, and 90.60% ± 0.40%, respectively, indicating that GCDD achieves stable retrieval performance and is not highly sensitive to random seeds.

(2) Other benchmark evaluations. To further evaluate the generalization ability of GCDD, we conduct experiments on ICFG-PEDES and RSTPReid, as reported in Table 3 and Table 4, respectively. These two datasets provide complementary evaluation settings and help examine whether GCDD can maintain effectiveness beyond CUHK-PEDES. On ICFG-PEDES, GCDD achieves 56.26%, 76.81%, and 83.02% on Rank-1, Rank-5, and Rank-10, respectively, outperforming all listed methods. On RSTPReid, GCDD obtains 52.20%, 75.40%, and 83.70%, achieving the best Rank-10 result while remaining competitive on Rank-1 and Rank-5. These results indicate that GCDD maintains stable and competitive performance under different benchmark settings. The two encoder variants further provide useful evidence for the robustness and adaptability of the proposed framework. Ours-IN-ViT replaces the CLIP visual encoder with an ImageNet-pretrained ViT and still achieves competitive results compared with several non-CLIP-based methods. This suggests that the proposed DTS, GFA, and QDA modules are not strictly tied to the CLIP visual backbone and can still enhance representations under an alternative visual encoder. Ours-CLIP-T replaces the BERT text encoder with the original CLIP text encoder while keeping the remaining framework unchanged. This variant also obtains competitive performance on both datasets, indicating that the proposed framework can be instantiated with different textual encoders. Nevertheless, the full CLIP-BERT-based GCDD consistently performs better than Ours-CLIP-T. Specifically, GCDD improves over Ours-CLIP-T by 0.78%, 2.23%, and 2.20% on ICFG-PEDES, and by 3.05%, 0.65%, and 0.65% on RSTPReid in terms of Rank-1, Rank-5, and Rank-10, respectively. These results suggest that, after downstream contrastive re-alignment, the BERT-based text encoder can provide more effective fine-grained textual modeling for attribute-rich pedestrian descriptions than the original CLIP text encoder. Overall, these results further demonstrate the generalization ability and robustness of GCDD, while also supporting the task-oriented encoder design of the proposed framework.

In summary, the comparison results demonstrate that GCDD achieves strong and competitive performance across different TI-ReID benchmarks. The consistent improvements on ICFG-PEDES and the competitive results on RSTPReid indicate that the proposed method is not limited to a single dataset but shows good generalization ability. Meanwhile, the results of Ours-IN-ViT and Ours-CLIP-T further verify the robustness of the proposed enhancement framework under different encoder configurations. Although the two variants also achieve competitive performance, the full CLIP-BERT-based GCDD remains the most stable and effective configuration, supporting the task-oriented design of the proposed framework.

### 4.2. Ablation Studies

To further examine the effectiveness of the proposed design, we conduct ablation and additional analysis experiments on CUHK-PEDES. This section analyzes the contributions of DTS, GFA, and QDA, compares different fusion strategies, evaluates computational cost, and studies the sensitivity of the DTS token retention ratio. These results provide a comprehensive validation of the proposed framework from the perspectives of component contribution, efficiency, and parameter robustness.

**Figure 6:**
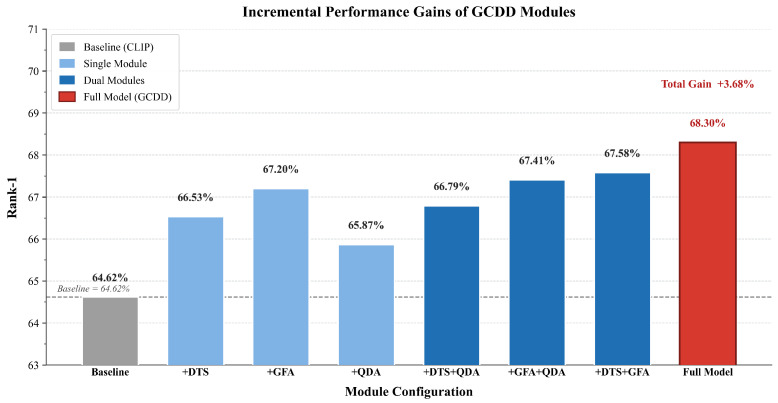
Incremental performance analysis on the CUHK-PEDES dataset.

**Figure 7:**
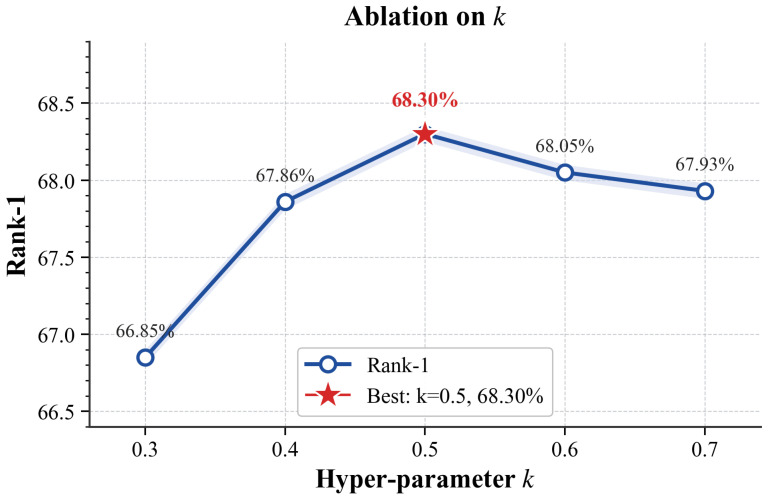
Parameter analysis of the DTS candidate retention ratio *k* on CUHK-PEDES. The same ratio is applied to both image and text branches.

(1) Component Ablation. This subsection evaluates the contribution of each component in the proposed GCDD framework. We use a dual-tower CLIP-ViT-B/16 and BERT-base model trained with the InfoNCE loss as the baseline. All ablation experiments are conducted on the CUHK-PEDES dataset, and the results in Table 5 show that each individual module brings consistent improvements over the baseline, demonstrating the effectiveness of the proposed design. Specifically, DTS improves Rank-1 from 64.62% to 66.53%, indicating that the hard token filtering strategy can suppress less informative or noisy tokens and enhance identity-related cues. GFA achieves the largest single-module Rank-1 gain, improving Rank-1 to 67.20%. This suggests that global-guided local feature refinement is particularly useful for improving semantic consistency while preserving useful local details. QDA also improves the baseline performance by introducing query-driven attentive aggregation, which helps the model aggregate more discriminative local cues into a compact retrieval-oriented representation. Although the individual gain of QDA is smaller than that of DTS and GFA, it still provides complementary information for final matching. The multi-module results further demonstrate the complementarity among DTS, GFA, and QDA. Among the two-module variants, DTS+GFA achieves the best overall performance, reaching 67.58%, 84.11%, and 90.54% on Rank-1, Rank-5, and Rank-10, respectively. This shows that token-level denoising and global-guided feature refinement can work effectively together. When all three modules are jointly integrated, the full GCDD model obtains the best results of 68.30%, 85.56%, and 91.03%. Compared with the baseline, it brings absolute gains of 3.68%, 2.59%, and 2.49% on Rank-1, Rank-5, and Rank-10, respectively. Figure 6 provides a visual comparison of the incremental gains introduced by each component. These results further demonstrate the complementary nature of DTS, GFA, and QDA, whose joint integration leads to improved retrieval performance.

(2) Fusion strategy ablation. To evaluate the effectiveness of the adopted fusion strategy, we compare several alternatives for combining the outputs of DTS, GFA, and QDA, including learnable static weights, sample-wise dynamic weights, attention-based fusion, arithmetic mean with branch pre-normalization, and the adopted arithmetic mean with post-fusion normalization. As reported in Table 6, different fusion strategies show different preferences across evaluation metrics. Sample-wise dynamic weighting achieves the highest Rank-5 accuracy, while branch pre-normalized averaging obtains the highest Rank-1 accuracy. However, neither strategy consistently outperforms the others across all evaluation metrics. In comparison, the adopted arithmetic mean with post-fusion normalization achieves competitive Rank-1 and Rank-5 performance and the best Rank-10 result, while introducing no additional fusion parameters. This suggests that the simple parameter-free averaging strategy provides a favorable balance between retrieval performance, stability, and parameter efficiency. Therefore, we adopt the arithmetic mean with post-fusion normalization as the default fusion strategy in GCDD.

(3) Computational Cost Analysis. To assess the efficiency of GCDD, we compare its computational cost with the baseline and representative methods, as reported in Table 7. This analysis examines whether the performance improvement of GCDD is achieved at the expense of excessive computational overhead. Although GCDD introduces more parameters and a larger model size than the baseline due to the additional DTS, GFA, and QDA branches, its inference FLOPs and estimated training FLOPs remain within a reasonable range. This is because the proposed enhancement modules operate within each modality and do not introduce expensive token-level cross-modal interactions during inference. Compared with TIPCB, GCDD requires fewer inference FLOPs and estimated training FLOPs while achieving higher Rank-1 accuracy, indicating a better trade-off between retrieval accuracy and computational efficiency. Compared with CFine, which adopts a similar CLIP-ViT-B/16 and BERT backbone, GCDD obtains slightly better retrieval performance with slightly lower inference and training FLOPs. These results suggest that the performance gain of GCDD is not simply obtained by increasing computational cost or model complexity, but mainly benefits from the proposed discriminative denoising and feature enhancement design. Overall, although GCDD is not the smallest model in terms of parameter size, it maintains the efficiency advantage of the dual-tower retrieval paradigm and achieves a favorable accuracy-efficiency trade-off through efficient intra-modal enhancement modules.

(4) Parameter Analysis. We further investigate the sensitivity of the candidate retention ratio in the DTS module. This analysis is important because the retention ratio directly determines how many local tokens are allowed to enter the subsequent fine-grained screening stage, thereby affecting the balance between discriminative information preservation and noise suppression. Specifically, $K_{ratio}^{img}$ and $K_{ratio}^{txt}$ are varied from 0.3 to 0.7, while all other settings are kept unchanged. Since the same ratio is applied to both image and text branches in this analysis, we use *k* to denote the candidate retention ratio for simplicity. As shown in Figure 7, the Rank-1 accuracy increases from 66.85% to 68.30% when *k* increases from 0.3 to 0.5, and then slightly decreases as *k* is further enlarged to 0.6 and 0.7. This trend reflects the trade-off between information preservation and noise suppression in DTS. When the retention ratio is too small, some identity-related tokens may be discarded at the preliminary screening stage, leading to insufficient discriminative information for cross-modal matching. In contrast, when the ratio is too large, more redundant or noisy tokens may be retained, weakening the denoising effect of DTS and reducing the discriminability of the learned representation. The best performance is obtained at k=0.5, suggesting that retaining approximately half of the tokens before adaptive thresholding provides a favorable balance between preserving useful identity cues and suppressing irrelevant information. Therefore, k=0.5 is adopted as the default setting in our experiments.

### 4.3. Qualitative Results

**Figure 8:**
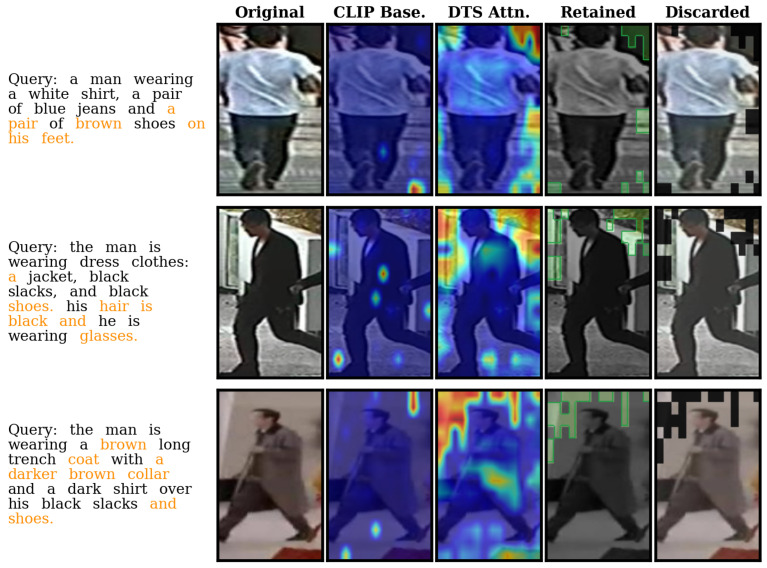
Visualization of DTS token selection. Orange words denote retained discriminative textual cues. Green-highlighted regions indicate retained visual patches, while darkened or masked regions indicate discarded patches. The results show that DTS preserves identity-related cues such as clothing, shoes, bags, and glasses, while suppressing background clutter and weakly informative regions.

To further analyze the denoising behavior of the proposed DTS module, we visualize the retained and discarded tokens in both textual and visual modalities. As shown in Figure 8, the orange words in the text descriptions denote the discriminative textual tokens or phrase-level cues retained by DTS. These cues mainly correspond to identity-related pedestrian attributes, such as clothing colors, shoes, bags, glasses, coats, and other fine-grained appearance details. For the visual modality, we present the original image, the CLIP baseline attention map, the DTS attention map, the retained visual patches, and the discarded visual patches. In the Retained column, green-highlighted regions indicate the image patches selected by DTS as informative cues for cross-modal matching, whereas in the Discarded column, the darkened or masked regions indicate patches removed due to their relatively low discriminative contribution. Compared with the CLIP baseline attention maps, DTS produces more selective and identity-focused responses, concentrating on pedestrian-centric regions and fine-grained attribute details while suppressing background clutter and visually less informative areas. These observations provide qualitative evidence that DTS performs token-level denoising by explicitly retaining discriminative textual and visual cues and discarding weakly informative tokens.

**Figure 9:**
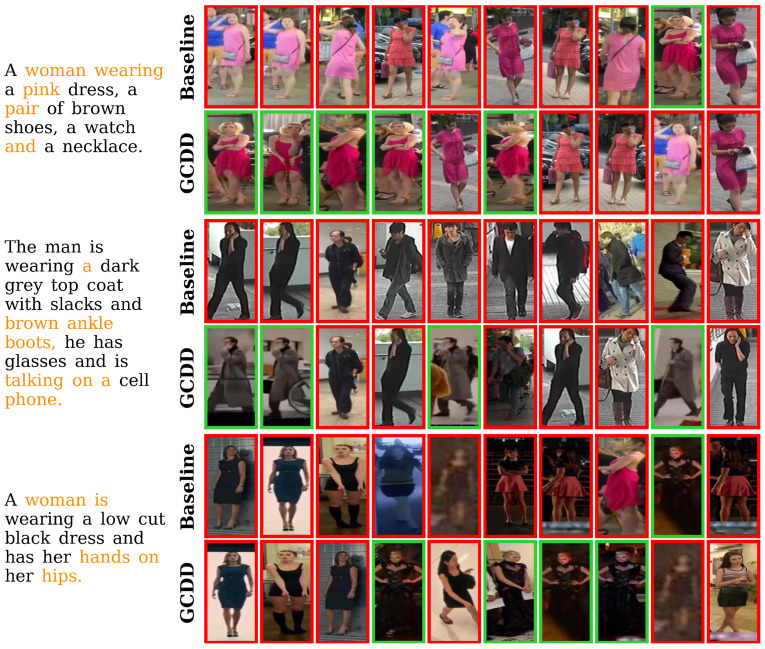
Qualitative comparison of the top-10 retrieval results produced by the Baseline (first row) and the proposed GCDD (second row) on the CUHK-PEDES dataset. The words highlighted in orange in the query text correspond to highly discriminative fine-grained cues identified by the DTS module. Green and red bounding boxes indicate correct and incorrect matches, respectively.

The qualitative retrieval results in Figure 9 present the top-10 matches returned by the baseline method and GCDD for three text queries. Overall, GCDD retrieves more relevant samples, with correct matches appearing more frequently at higher ranks. This advantage becomes more pronounced when the query contains multiple fine-grained attributes, such as clothing color, accessories, and pose. In contrast, the baseline often returns samples that match only part of the description. For instance, some retrieved images share a similar clothing color but fail to satisfy other important details specified in the text. The baseline is also more susceptible to background clutter and visual ambiguity, which may lead to mismatched results among the top-ranked samples. By contrast, GCDD is able to retrieve images that better correspond to the complete textual description, even in visually complex scenes. These observations indicate that GCDD achieves more accurate fine-grained alignment between visual content and textual semantics. In addition, the highlighted keywords suggest that informative textual cues play a crucial role in accurate retrieval. These qualitative findings are also consistent with the design motivations of DTS, GFA, and QDA, which aim to emphasize discriminative textual information and enhance visual-text semantic matching.

## 5. Conclusions

In this work, we propose GCDD, a dual-tower framework with three complementary feature enhancement branches for TI-ReID. Our objective is to more effectively exploit CLIP-based cross-modal priors for improved alignment and robust representation learning. Specifically, the Discriminative Token Selection (DTS) module adopts a two-stage filtering strategy to suppress background noise while preserving highly informative visual and textual tokens. The Global-Guided Feature Adaptation (GFA) module recalibrates local features under modality-specific global semantic guidance, enhancing cross-granularity semantic consistency. The Query-Driven Aggregation (QDA) module further constructs a retrieval-oriented global representation via query-driven attentive pooling. The outputs of the three branches are fused through a parameter-free averaging strategy, yielding enhanced modality-specific representations while maintaining compatibility with the standard dual-tower inference paradigm. Extensive experiments on three public benchmark datasets demonstrate the effectiveness and competitiveness of the proposed method.

## 6. Limitations and Future Work

Although GCDD achieves competitive performance on three TI-ReID benchmarks, several limitations remain. First, the additional DTS, GFA, and QDA branches increase the model parameter size, although the overall computational cost remains reasonable. Future work will explore more lightweight designs for resource-constrained applications. Second, DTS currently depends on a predefined token retention ratio, and more adaptive token selection strategies may further improve robustness across datasets and complex scenarios. Finally, although this work focuses on TI-ReID, the proposed modules operate on modality-specific token sequences and are not inherently limited to pedestrian retrieval. They may be extended to other fine-grained cross-modal retrieval tasks, such as text-to-image retrieval on CUB or FashionIQ, and adapted to other vision-language backbones. Systematic validation on more datasets and backbone architectures will be considered in future work. 

## Figures and Tables

**Table 1:** Statistics of the TI-ReID datasets used in our experiments.

Dataset	Identities			Images			Total Texts	Texts / Img	Avg. Len
	Train	Val	Test	Train	Val	Test			
CUHK-PEDES	11,003	1000	1000	34,054	3078	3074	80,412	2	≥23
ICFG-PEDES	3102	–	1000	34,674	–	19,848	54,522	1	37
RSTPReid	3701	200	200	18,505	1000	1000	41,010	2	∼23

**Table 2:** Comparison with other methods on CUHK-PEDES.

Method	Architecture	Rank-1	Rank-5	Rank-10
Dual Path	RN50-RN50	35.44	59.59	70.39
CMPM	RN50-LSTM	44.02	69.06	78.37
DSSL	RN50-biGRU	57.99	77.06	84.95
SSAN	RN50-LSTM	61.47	81.44	87.26
TIPCB	RN50-BERT	63.32	82.92	89.04
CFine	CLIP-BERT	68.15	84.73	90.25
CSKT	CLIP-CLIP	**69.05**	84.93	90.25
Ours-IN-ViT	IN-ViT-BERT	62.10	82.41	88.01
Ours-CLIP-T	CLIP-CLIP	66.38	80.54	86.81
GCDD	CLIP-BERT	68.30	**85.56**	**91.03**

Note: Bold values indicate the best performance.

**Table 3:** Comparison with other methods on ICFG-PEDES.

Method	Architecture	Rank-1	Rank-5	Rank-10
Dual Path	RN50-RN50	32.48	52.03	61.55
CMPM	RN50-LSTM	34.14	57.76	67.30
DSSL	RN50-biGRU	42.55	64.42	73.21
SSAN	RN50-LSTM	51.76	70.74	78.04
TIPCB	RN50-BERT	52.14	73.11	80.35
CFine	CLIP-BERT	55.43	75.44	81.42
CSKT	CLIP-CLIP	54.38	75.86	82.65
Ours-IN-ViT	IN-ViT-BERT	49.88	69.95	77.44
Ours-CLIP-T	CLIP-CLIP	55.48	74.58	80.82
GCDD	CLIP-BERT	**56.26**	**76.81**	**83.02**

Note: Bold values indicate the best performance.

**Table 4:** Comparison with other methods on RSTPReid.

Method	Architecture	Rank-1	Rank-5	Rank-10
Dual Path	RN50-RN50	33.30	62.20	73.75
CMPM	RN50-LSTM	39.10	65.90	77.10
DSSL	RN50-biGRU	39.50	66.20	75.25
SSAN	RN50-LSTM	42.15	66.80	75.80
TIPCB	RN50-BERT	45.95	71.75	81.05
CFine	CLIP-BERT	49.05	72.80	82.20
CSKT	CLIP-CLIP	**54.30**	**76.50**	83.48
Ours-IN-ViT	IN-ViT-BERT	44.50	70.75	79.55
Ours-CLIP-T	CLIP-CLIP	49.15	74.75	83.05
GCDD	CLIP-BERT	52.20	75.40	**83.70**

Note: Bold values indicate the best performance.

**Table 5:** Ablation study of GCDD on CUHK-PEDES.

Model	Components			Metrics		
	DTS	GFA	QDA	Rank-1	Rank-5	Rank-10
0	**✗**	**✗**	**✗**	64.62	82.97	88.54
1	**✓**	**✗**	**✗**	66.53	84.13	89.37
2	**✗**	**✓**	**✗**	67.20	83.22	89.54
3	**✗**	**✗**	**✓**	65.87	83.42	88.80
4	**✓**	**✗**	**✓**	66.79	83.13	88.81
5	**✗**	**✓**	**✓**	67.41	83.33	90.49
6	**✓**	**✓**	**✗**	67.58	84.11	90.54
7 (Ours)	**✓**	**✓**	**✓**	**68.30**	**85.56**	**91.03**

Note: **✓** and **✗** indicate used and unused components, respectively. Bold values indicate the best performance.

**Table 6:** Ablation study of different fusion strategies on CUHK-PEDES.

Fusion Strategy	Rank-1	Rank-5	Rank-10	Add. Params
Learnable Static Weights	67.95	85.07	90.39	3
Sample-wise Dynamic Weights	68.20	**86.10**	90.54	1.18 M
Attention-based Fusion	67.74	84.67	89.88	2.36 M
Arithmetic Mean (branch pre-norm)	**68.75**	84.68	90.24	0
Arithmetic Mean (Ours)	68.30	85.56	**91.03**	0

Note: Bold values indicate the best performance.

**Table 7:** Computational cost comparison with matched analog methods on CUHK-PEDES.

Method	Params (M)	Size (MB)	Infer. FLOPs (G)	Est. Train FLOPs (G)	Rank-1
Baseline	195.28	744.94	23.41	119.58	64.62
SSAN	82.65	315.30	17.68	90.31	61.47
TIPCB	184.75	704.76	43.86	224.04	63.32
CFine	204.74	781.01	27.16	138.72	68.15
GCDD (Ours)	245.28	935.66	26.73	136.56	**68.30**

Note: Bold values indicate the best performance.

## Data Availability

The datasets used in this study are publicly available from their official sources, including CUHK-PEDES, ICFG-PEDES, and RSTPReid.
